# Direct Injection of CRISPR/Cas9-Related mRNA into Cytoplasm of Parthenogenetically Activated Porcine Oocytes Causes Frequent Mosaicism for Indel Mutations

**DOI:** 10.3390/ijms160817838

**Published:** 2015-08-03

**Authors:** Masahiro Sato, Miyu Koriyama, Satoshi Watanabe, Masato Ohtsuka, Takayuki Sakurai, Emi Inada, Issei Saitoh, Shingo Nakamura, Kazuchika Miyoshi

**Affiliations:** 1 Section of Gene Expression Regulation, Frontier Science Research Center, Kagoshima University, Kagoshima 890-8544, Japan; 2 Laboratory of Animal Reproduction, Faculty of Agriculture, Kagoshima University, Kagoshima 890-0065, Japan; E-Mails: k3326891@kadai.jp (M.K.); kmiyoshi@agri.kagoshima-u.ac.jp (K.M.); 3 Animal Genome Research Unit, Division of Animal Science, National Institute of Agrobiological Sciences, Ibaraki 305-8602, Japan; E-Mail: kettle@affrc.go.jp; 4 Division of Basic Molecular Science and Molecular Medicine, School of Medicine, Tokai University, Kanagawa 259-1193, Japan; E-Mail: masato@is.icc.u-tokai.ac.jp; 5 Department of Cardiovascular Research, Graduate school of Medicine, Shinshu University, Nagano 390-8621, Japan; E-Mail: tsakurai@shinshu-u.ac.jp; 6 Department of Pediatric Dentistry, Graduate School of Medical and Dental Sciences, Kagoshima University, Kagoshima 890-8544, Japan; E-Mail: inada@dent.kagoshima-u.ac.jp; 7 Division of Pediatric Dentistry, Department of Oral Health Sciences, Course for Oral Life Science, Graduate School of Medical and Dental Sciences, Niigata University, Niigata 951-8514, Japan; E-Mail: isaito@dent.niigata-u.ac.jp; 8 Division of Biomedical Engineering, National Defense Medical College Research Institute, Saitama 359-8513, Japan; E-Mail: snaka@ndmc.ac.jp

**Keywords:** α-Gal epitope, α-1,3-galactosyltransferase, CRISPR/Cas9, microinjection, mRNA, mosaicism, biallelic KO, indel mutations, isolectin BS-I-B_4_

## Abstract

Some reports demonstrated successful genome editing in pigs by one-step zygote microinjection of mRNA of CRISPR/Cas9-related components. Given the relatively long gestation periods and the high cost of housing, the establishment of a single blastocyst-based assay for rapid optimization of the above system is required. As a proof-of-concept, we attempted to disrupt a gene (*GGTA1*) encoding the α-1,3-galactosyltransferase that synthesizes the α-Gal epitope using parthenogenetically activated porcine oocytes. The lack of α-Gal epitope expression can be monitored by staining with fluorescently labeled isolectin BS-I-B_4_ (IB4), which binds specifically to the α-Gal epitope. When oocytes were injected with guide RNA specific to *GGTA1* together with enhanced green fluorescent protein (*EGFP*) and human *Cas9* mRNAs, 65% (24/37) of the developing blastocysts exhibited green fluorescence, although almost all (96%, 23/24) showed a mosaic fluorescent pattern. Staining with IB4 revealed that the green fluorescent area often had a reduced binding activity to IB4. Of the 16 samples tested, six (five fluorescent and one non-fluorescent blastocysts) had indel mutations, suggesting a correlation between EGFP expression and mutation induction. Furthermore, it is suggested that zygote microinjection of mRNAs might lead to the production of piglets with cells harboring various mutation types.

## 1. Introduction

Gene modification using homologous recombination (HR)-based gene targeting is a powerful technique for exploring gene function and producing human disease models. HR in cultured embryonic stem (ES) cells is employed to generate “targeted (KO, knockout)” ES cells, which will then be transplanted into the blastocoel of host embryos to produce germ-line competent chimeric mice. However, this technology has been difficult to apply to large animals, such as swine, sheep, and cattle, since ES cells from these animals that enable the production of chimeric animals with germ-line competence have not been available. Therefore, until recently, the production of KO somatic cells, such as embryonic fibroblasts, and the subsequent somatic cell nuclear transfer (SCNT) of these cells to enucleated oocytes has been the only method available for the production of KO pigs and cattle [1,2]. However, the generation of biallelic KO cell lines using HR is inefficient and the process to obtain biallelic KO animals through breeding is very time-consuming.

Recently, gene knockout was successfully achieved by employing newly developed genome editing techniques using zinc-finger nuclease (ZFN), transcription activator-like effector nucleases (TALEN), and clustered regularly interspaced short palindromic repeats (CRISPR)/CRISPR-associated (Cas) (CRISPR/Cas9) [3,4]. In the case of CRISPR/Cas9-based genome editing, it requires a guide RNA (gRNA) which can bind to the specific chromosomal DNA site together with the Cas9 endonuclease [5,6,7,8]. Once bound, two independent nuclease domains in Cas9 will each cleave one of the DNA strands’ three bases upstream of the protospacer adjacent motif (PAM), introducing double-strand breaks (DSBs) at the target site of the host chromosome, which are repaired by non-homologous end-joining (NHEJ). The NHEJ-based repair process generates the insertion or deletion of nucleotides, meaning an indel mutation, which causes a frame-shift that alters the encoded proteins or the formation of a premature stop codon and, finally, leads to the generation of a loss-of-function allele. Using the CRISPR/Cas9 system, successful genome modifications have been achieved in various organisms, including *Drosophila* [9,10,11], *Caenorhabditis elegans* [12], zebrafish [13,14,15], mice [16,17,18,19,20,21], rats [22,23,24,25], rabbits [26], pigs [27,28,29], cattle [30], monkeys [31], and humans [32,33,34,35,36]. Owing to the difficulty in design and assembly, and the limited availability of target sites [37], CRISPR/Cas9 is employed more frequently than the ZFN- and TALEN-based methods in the production of genetically modified animals [3,4].

The CRISPR/Cas9 system-mediated genome editing in pigs has not been extensively studied. Sato *et al.* [27] recently demonstrated that transfection of porcine embryonic fibroblasts (PEFs) with CRISPR/Cas9-related DNA resulted in the generation of cells with a biallelic KO phenotype. Furthermore, somatic cell nuclear transfer (SCNT) of these KO cells as donors led to the production of cloned blastocysts incapable of expressing the target gene product [27]. Hai *et al.* [28] demonstrated that one-step microinjection of mRNA of CRISPR/Cas9-related components into the cytoplasm of zygotes isolated from oviducts could lead to the production of biallelic KO piglets. More recently, Whitworth *et al.* [29] also provided similar results using *in vitro*-produced zygotes. These findings are encouraging and have enabled the production of genetically modified piglets through one-step injection of CRISPR/Cas9-related components (mRNAs) into zygotes. However, researchers must validate whether the above-mentioned components are operative in pig tissues. For CRISPR/Cas9-mediated genome editing system optimization, many researchers have used cultured cells, but it is not known whether the data obtained using this system can be applied *in vivo*. Given the relatively long gestation periods and the high cost of housing, testing CRISPR/Cas9-induced mutations using the genome of newborn piglets is challenging. Therefore, it may be required to establish a single embryo-based assay for detecting such mutations at the protein and gene levels, which will enable researchers to test whether the CRISPR/Cas9-related components constructed are effective for inducing mutations in the target locus. Blastocysts, stages just prior to implantation, are appropriate materials to test this issue since they can successfully be used for PCR-based amplification for molecular biological analysis [38,39] and express many types of mRNAs, including α-1,3-galactosyltransferase (α-GalT) gene (*GGTA1*) [40]. It takes seven days to obtain blastocysts after fertilization in pigs. Therefore, rapid evaluation of CRISPR/Cas9-related components is possible if these are used as starting materials. Moreover, it is easy to monitor the consequence of loss-of-gene function during the preimplantation stages at a single embryo level if appropriate antibodies or lectins specifically reactive to the cell-surface molecules expressed in those embryos are available.

In order to establish a single blastocyst-based assay in pigs, we selected *GGTA1* as the targeted integration site. *GGTA1* can synthesize an α-Gal epitope (Galα1-3Galβ1-4GlcNAc-R) that is easily recognizable by staining the cells with the α-Gal epitope-specific isolectin, BS-I-B_4_ (IB4) [41]. Thus, it is easy to monitor *GGTA1* expression in embryos by staining them with fluorescently labeled IB4. In fact, our previous data revealed that porcine cells with the biallelic KO phenotype caused by CRISPR/Cas9-mediated indel mutations are not stained with fluorescently labeled IB4 [27]. We prepared guide RNA specific to exon 4 (containing the ATG site) of *GGTA1*, which has been proven to be effective during CRISPR/Cas9-meditated gene disruption [27], together with mRNAs for enhanced green fluorescent protein (EGFP; used for monitoring the fate of cytoplasmically injected mRNA) and human Cas9. These were microinjected into the cytoplasm of parthenogenetically activated (PA) porcine oocytes to examine the mode of action of CRISPR/Cas9-mediated gene disruption in porcine parthenotes in terms of loss of α-Gal epitope expression. Furthermore, we employed whole genome amplification (WGA) which potentially allows multiple PCR-based analyses from the small amount of genomic DNA isolated from a single blastocyst [39,42].

## 2. Results and Discussion

### 2.1. Evaluation of CRISPR/Cas9-Based Genome Editing Ability in Porcine Embryos

**Figure 1:**
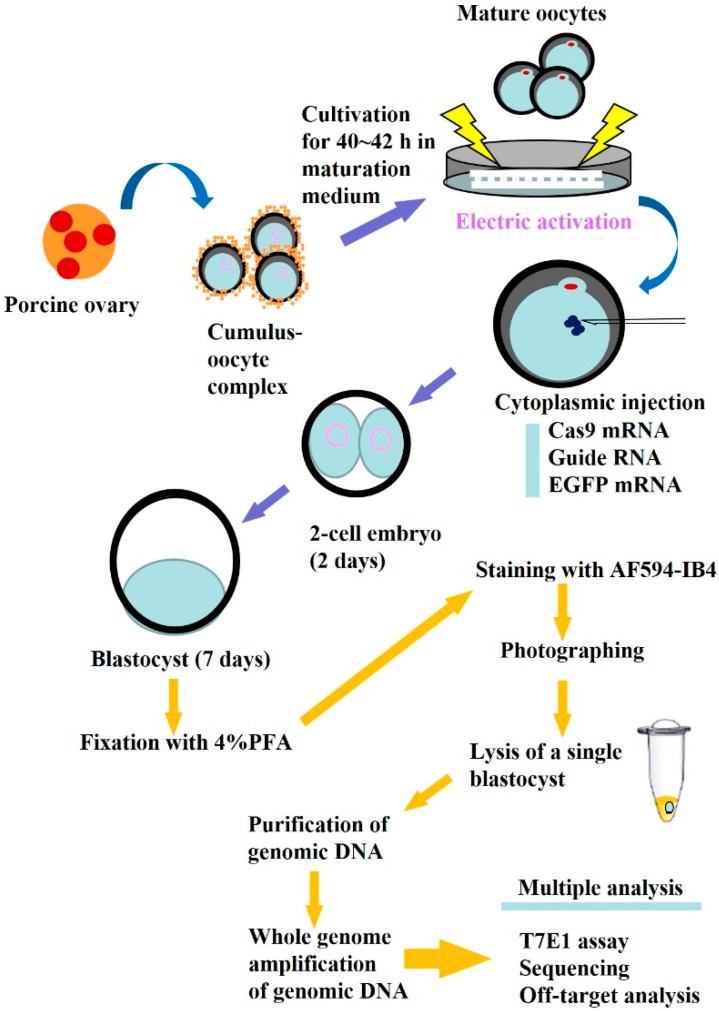
Schematic representation of a single blastocyst-based assay after isolation of porcine oocytes from an ovary, *in vitro* maturation, electric activation, and subsequent cytoplasmic injection with CRISPR/*Cas9*-related mRNA and *EGFP* mRNA.

We chose to use porcine PA oocytes that are able to develop *in vitro* at least up to blastocysts, as do normally fertilized oocytes [43]. The experimental procedure in this study is outlined in Figure 1. After electric activation of *in vitro*-matured oocytes, these were cytoplasmically injected with a solution (~2 pL) containing *in vitro* synthesized *hCas9* mRNA (2 ng/μL), gRNA (2 ng/μL; specific to *GGTA1* exon 4), and *EGFP* mRNA (2 ng/μL) and were then cultured *in vitro* until blastocyst formation for approximately seven days. From a total of 107 oocytes injected, both the cleavage rate (79.4%) and blastocyst formation rate (34.6%) were comparable to those of intact PA oocytes (67.7% and 33.1% for cleavage and blastocyst formation, respectively) described in a previous article [44]. Thus, the concentration of RNA used does not seem to be harmful for porcine embryonic development.

**Figure 2:**
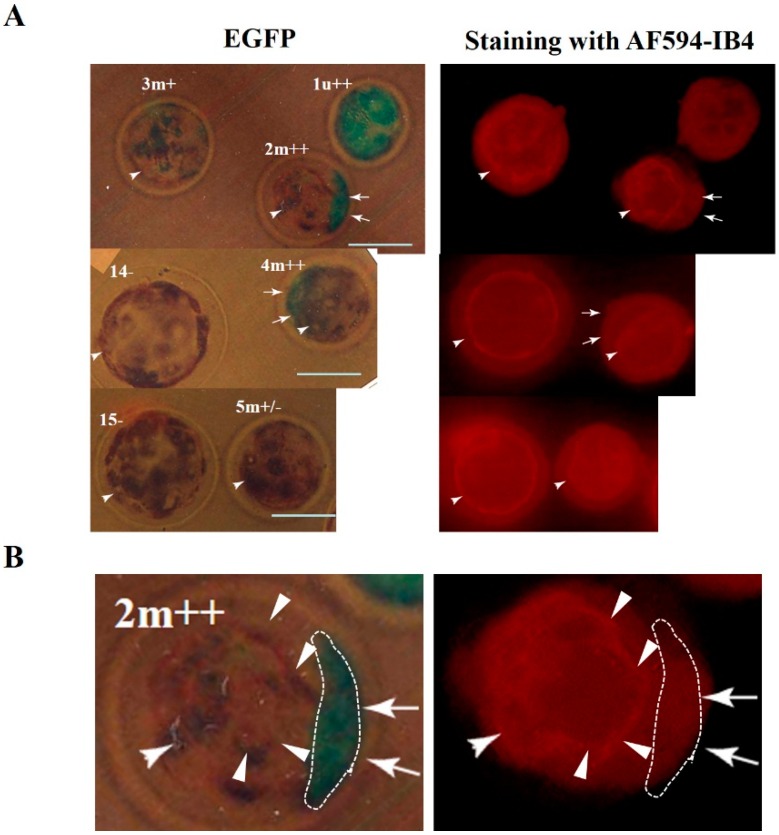
(**A**) Fluorescent images of single blastocysts (1u++, 2m++, 3m+, 4m++, 5m+/−, 14−, and 15−) developed after oocyte injection with CRISPR/*Cas9*-related mRNA and *EGFP* mRNA. Blastocysts were fixed for a short period, stained with AF594-IB4, and then inspected for EGFP- and AF594-derived fluorescence under a fluorescence microscope. u++, uniform and strong fluorescence; m++, mosaic and strong fluorescence; m+, mosaic and moderate fluorescence; m+/−, mosaic and slight fluorescence; −, no fluorescence. Bar = 100 μm; and (**B**) Magnified images of the 2m++ blastocyst shown in (**A**). The area enclosed by the dotted line shows EGFP-derived fluorescence and reduced α-Gal epitope expression (arrows). The other non-fluorescent area within the same embryos was distinctly stained with lectin, showing a ring-like staining pattern (arrowheads).

After brief fixation in 4% paraformaldehyde (PFA) of all of the developing blastocysts, they were stained with Alexa Fluor (AF) 594-labeled IB4 (AF594-IB4) to check α-Gal epitope expression. Twenty-four of the 37 (64.9%) blastocysts derived from the mRNA-injected oocytes exhibited EGFP-derived fluorescence, but almost all of these (23/24) showed a mosaic fluorescent pattern. The data from blastocysts obtained from one course of injection are shown in Table 1. In the left panel of Figure 2A, typical examples are shown. Of the five fluorescent embryos, one embryo (labeled 1u++) exhibits green fluorescence in the entire embryo, while the other remaining four embryos (2m++, 3m+, 4m++, and 5m+/−) do not. The other two embryos (14− and 15−) do not show a fluorescent signal (left panel of Figure 2A). Staining with AF594-IB4 revealed that all embryos were stained, fluorescent as well as non-fluorescent embryos (right panel of Figure 2A). Notably, reduced AF594-IB4 staining was observed in the entire 1u++ embryo, although a greater magnification failed to discriminate the difference in red fluorescence between the stained embryo and the other non-fluorescent embryos (embryos 14− and 15− in the right panel of Figure 2A). The reduced AF594-IB4 staining was also seen in some embryonic portions that showed green fluorescence (indicated by arrows in embryos 2m++ and 4m++ of Figure 2A). In contrast, the other non-fluorescent area within the same embryos was distinctly stained with lectin, showing a ring-like staining pattern (indicated by arrowheads in embryos 2m++ and 4m++ of Figure 2A). In Figure 2B, in which the embryo 2m++ shown in Figure 2A is further magnified, the green fluorescent area enclosed by a dotted line showed weaker AF594-IB4 staining, indicating a correlation between the green fluorescent signal and reduced α-Gal epitope expression. These findings suggest that several porcine blastocysts derived from CRISPR/Cas9 mRNA-injected oocytes are comprised of at least three types of cells, biallelic KO (showing complete loss of α-Gal epitope expression), monoallelic KO (showing α-Gal epitope expression), and wild-type cells (showing α-Gal epitope expression).

**Table 1:** Summary of the properties of blastocysts derived from parthenogenetically activated (PA) oocytes injected with CRISPR/*Cas9*-related mRNA + *EGFP* mRNA ^a^.

Name of Blastocysts	Fluorescence Observation after Staining with AF594-IB4 EGFP-Derived		Production of ~350 bp Fragment ^d^ AF594-Derived	Indel Mutations after T7E1 Assay ^e^
	Fluorescence ^b^	Fluorescence ^c^		
1	u+	Totally decrease	OK	Yes
2	m++	Mosaic	OK	Yes
3	m+	Mosaic	OK	Yes
4	m++	Mosaic	OK	Yes
5	m+/−	Uniform	OK	No
6	m+/−	Uniform	OK	No
7	m+/−	Uniform	OK	No
8	m+/−	Uniform	OK	No
9	m+/−	Uniform	OK	No
10	m+	Mosaic	OK	Yes
11	−	Uniform	OK	Yes
12	−	Uniform	OK	No
13	−	Uniform	Insufficient	ND
14	−	Uniform	OK	No
15	−	Uniform	OK	No
16	−	Uniform	OK	No
17	−	Uniform	OK	No

^a^ Cytoplasmic injection of *Cas9* mRNA + gRNA (targeted to exon 4 of *GGTA1*) + *EGFP* mRNA are performed towards PA oocytes. They were then cultured for seven days until they formed blastocysts. These were then fixed, stained with AF594-IB4, and subjected to molecular analyses, as shown in the experimental process flow chart in Figure 1; ^b^ Blastocysts stained with AF594-IB4 were first inspected for EGFP-derived green fluorescence under a fluorescence microscope. Uniform and mosaic fluorescent signals are shown as “u” and “m”, respectively. The fluorescence strength was classified as ++ (strong fluorescence), + (moderate fluorescence), +/− (faint fluorescence), and − (no fluorescence); ^c^ AF594-derived red fluorescence was inspected under a fluorescence microscope. Blastocysts with some portions showing a decreased fluorescent signal were labeled as “mosaic” embryos. Blastocysts in which the entire embryo exhibited bright fluorescence were labeled as embryos showing “uniform” staining; ^d^ Blastocysts from which WGA-derived genomic DNA was used as template to produce ~350 bp fragments after the first and second round of PCR were labeled as “OK”; ^e^ Blastocysts which produced cleaved bands of ~100 and 150 bp after the T7E1 assay were labeled as “Yes”, while those without the cleaved bands were labeled as “No”; and ND, not determined.

**Figure 3:**
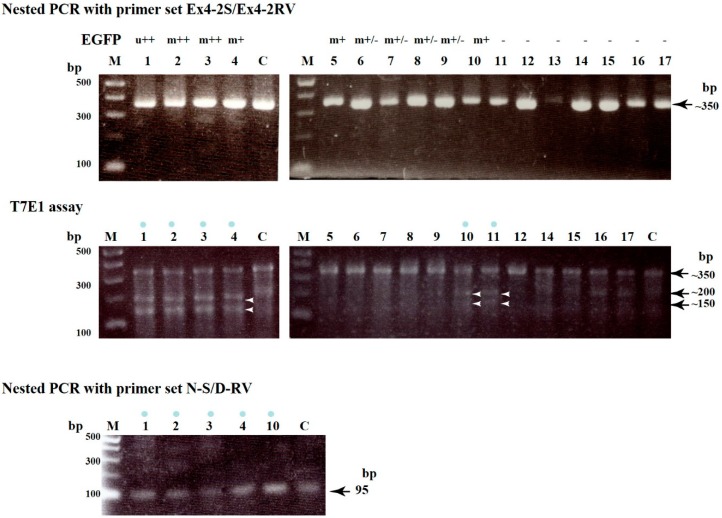
**Upper panel**. Production of ~350 bp products after first and nested PCR of genomic DNA initially amplified using WGA from a single blastocyst. Lanes **1**–**17** correspond to single numbered blastocysts (a part of these blastocysts is shown in Figure 2). The symbols above each lane indicate the fluorescence distribution type (ubiquitous (u) or mosaic (m)) and its strength (strong (++), moderate (+), slight (+/−), or none (−)); **Middle panel**: A T7E1-based assay for each single blastocyst. The approximately 350 bp PCR products shown in the upper panel were mixed with control (C) DNA at a ratio of 1:1, denatured, re-annealed, and then incubated with the T7E1 enzyme for 1 h at 37 °C. The resulting products were then electrophoresed in a 2% agarose gel. If samples have indel mutations, two fragments, namely ~200 and ~150 bp, were generated as cleaved products of the original ~350 bp products. The blue dots above the lanes indicate samples with mutations. Arrowheads indicate the position of cleaved bands, namely ~200 and 150 bp bands. Lanes **1**–**17** correspond to single numbered blastocysts as shown in the upper panel (except blastocyst number 13, which DNA amplification failed). Lane **C** indicates genomic DNA from normal PEFs used as a negative control; **Lower panel**: Production of 95 bp products after nested PCR of the first PCR products shown in the upper panel using primer sets N-S and D-RV (see Figure S1 and Table S1). Lanes **1**–**4** and **10** (all of which were identified as having mutations) correspond to single numbered blastocysts as shown in the upper and middle panels. Lane **C** indicates genomic DNA from normal porcine embryonic fibroblasts (PEFs) used as a positive control. **M**, 100 bp ladder markers.

Next, we performed molecular biological analyses to determine whether these fluorescent blastocysts possessed indel mutations at the target locus. Each fixed blastocyst was lysed and genomic DNA was isolated from the lysate. Before PCR-based amplification of the target locus, we employed WGA to amplify the whole genome of a single blastocyst. According to our previous data, it is possible to obtain ~84 ng of genomic DNA per blastocyst [42]. When PCR was performed using WGA products as a template, almost all of the samples (16/17; which include 10 fluorescent and seven non-fluorescent blastocysts) yielded distinct ~350 bp products corresponding to the region spanning the target site (upper panel of Figure 3, Table 1, Figure S1). In order to explore the possible presence of indel mutations in these amplified PCR products, we performed a T7E1-based cleavage assay. Of the 16 samples tested, six (five fluorescent and one non-fluorescent blastocysts) had two cleaved bands of ~200 and ~150 bp (middle panel of Figure 3), suggesting cleavage downstream of the ATG codon (translation initiation codon) of *GGTA1*.

**Figure 4:**
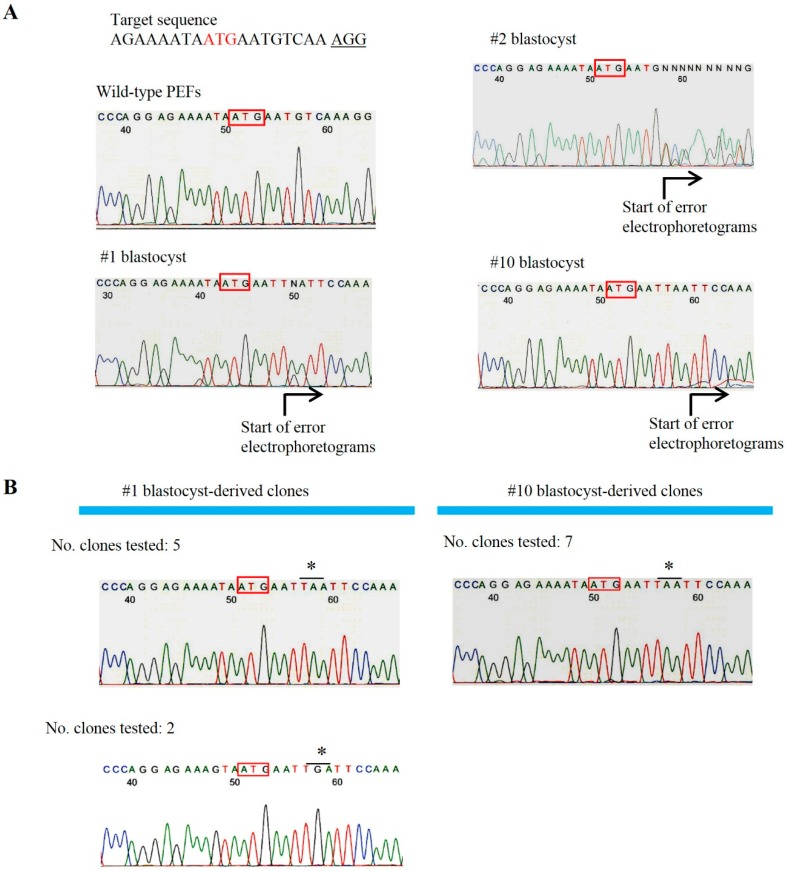
(**A**) Direct sequencing of PCR products (~350 bp) shown in the middle panel of Figure 3 from blastocysts No. 1, 2 and 10. Black arrows below the DNA sequence traces show overlapping peaks caused by additional indel mutations. Boxes indicate the translation initiation ATG codon; (**B**) Sequencing of the inserts derived from blastocysts no. 1 and 10 cloned into a TA cloning vector using the E-S primer set. Boxes indicate the translation initiation ATG codon. * indicate translation termination codons. In the above left corner of each DNA sequence trace, the number of clones tested is shown.

In order to examine in more detail where indel mutations occurred in *GGTA1*, the ~350 bp PCR products from blastocysts no. 1, 2, and 10 (all of which were identified as samples with indel mutations by the T7 endonuclease 1 (T7E1) assay; middle panel of Figure 3) together with the control samples (products from normal PEFs) were subjected to direct sequencing using the E-S primer set (corresponding to the sequence upstream of the ATG; Figure S1 and Table S1). As predicted, there were disordered DNA sequence traces downstream of the ATG codon in the experimental samples (indicated by the phrase “start of error electrophoretograms” in Figure 4A), indicating the presence of at least one or multiple mutations. In contrast, no disordered DNA sequence traces were noted in the control samples (labeled as “Wild-type PEFs” in Figure 4A). Some of these PCR products were cloned into a TA cloning vector and randomly picked *E. coli* transformants were sequenced using the E-S primer set. When clones carrying ~350 bp PCR products from blastocyst no. 1 were examined, at least two types of clones were identified (Figure 4B). Both types had a stop codon (TAA or TGA) downstream of the ATG codon that caused premature α-GalT protein synthesis termination. Unfortunately, we were unable to identify clones carrying the wild-type sequence. When clones carrying PCR products from blastocyst No. 10 were examined, only one type of clone that possessed a TAA downstream of the ATG codon was identified (Figure 4B). As in the clones derived from blastocyst No. 1, there were no clones carrying the wild-type allele.

The above sequencing data suggest that blastocysts no. 1 and 10 have no *GGTA1* wild-type allele. However, the previous staining data using AF594-IB4 (see Figure 2) clearly suggested that they should have a *GGTA1* wild-type allele. In order to explore this possibility, we performed a PCR using the ~350-bp PCR products derived from the samples with indel mutations (numbered 1, 2, 3, 4 and 10) with the N-S (corresponding to the normal sequence spanning the ATG codon of *GGTA1*) and D-RV primer sets (see Figure S1 and Table S1). The results are shown in the lower panel of Figure 3. All of the samples tested had a clear band (95 bp) corresponding to the wild-type allele. Sequencing of this fragment revealed that the resulting PCR products had a normal *GGTA1* sequence (data not shown). Therefore, blastocyst No. 1, which was identified as a ubiquitously EGFP-expressing embryo, might comprise at least three types of cells: wild-type cells, and bialellic and monoallelic KO cells. In this case, there were at least two different types of mutated alleles. As for blastocyst no. 10, which had been shown to have a mosaic EGFP-derived fluorescent signal pattern, it might also comprise three types of clones: wild-type cells, and bialellic and monoallelic KO cells. In this case, there was at least one type of mutated allele.

### 2.2. Uncovering Possible Off-Target Mutations in the CRISPR/Cas9-Related mRNA-Injected Porcine Embryos

**Figure 5:**
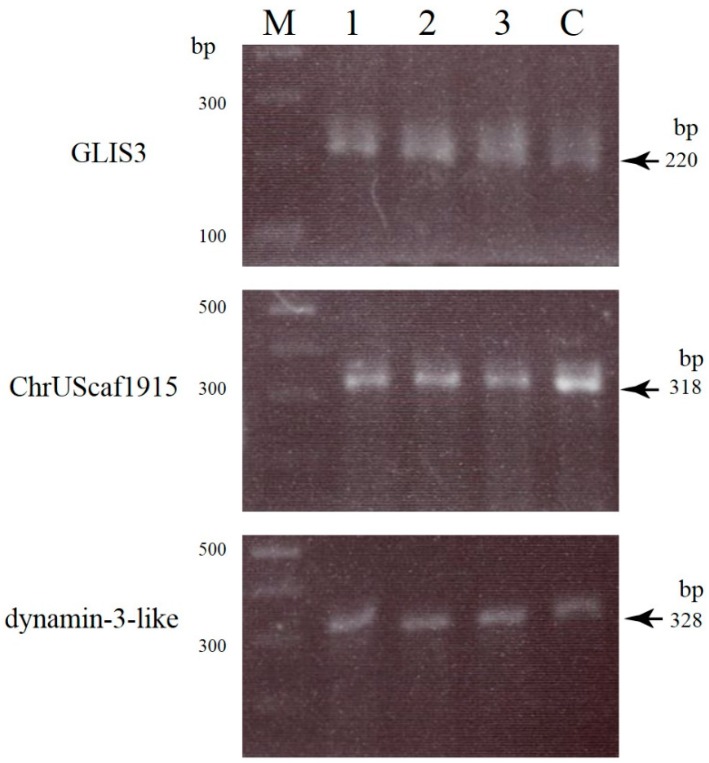
T7E1 analyses of three potential off-target sites (zinc finger protein *GLIS3*, *ChrUScaf1915*, and *dynamin-3-like*) in blastocysts no. 1 (lane **1**), 2 (lane **2**), and 10 (lane **3**) together with normal PEFs (lane **C**). Two bands (~140 and ~80 bp) are expected to be cleaved from the 220 bp PCR products of *GLIS3*. Two bands of ~200 and 120 bp are expected to be cleaved from the 318 bp PCR products of *ChrUScaf1915*. Two bands of ~200 and ~130 bp bands are expected to be cleaved from the 328 bp PCR products of *dynamic-3-like*. However, there is no appreciable off-target cleavage in the samples tested. **M**, 100 bp ladder markers.

It is known that CRISPR/Cas9-based genome editing often causes off-target mutations in irrelevant loci of certain cell types [45,46]. Therefore, we performed off-target analysis in blastocysts no. 1, 2, and 10, all of which had been identified as embryos with indel mutations. Based on the prediction method published in http://crispr.mit.edu, we selected three genes (zinc finger protein *GLIS3*, *ChrUScaf1915*, and *dynamin-3-like*; see Table S2) as potential off-target sites. These three genes are the top three high-scoring genes (ranging from 4.6 to 2.6; Table S2). A T7E1 assay demonstrated that no off-target cleavage was detected in the samples examined (Figure 5).

At present, direct injection of CRISPR/*Cas9*-related mRNA into fertilized eggs is thought to be a powerful technique for the production of biallelic KO animals, including mice, rats, rabbits, and pigs [16,17,18,19,22,23,24,25,26,28,29]. However, most studies have focused on the molecular biological analysis of the resulting fetuses or newborns to confirm the success of this genome editing system. This is a time-consuming and labor-intensive process in order to determine whether the gRNA constructed is suitable for enabling efficient KO of a target gene. Therefore, testing at earlier developmental stages has long been awaited. Indeed, Sakurai *et al.* [39] demonstrated that the T7E1 assay could successfully be used for detection of indel mutations in a single blastocyst. However, protein localization determination on the genome-edited embryos seemed challenging. The ideal approach would be the visualization of the KO effect by simple staining of a whole embryo by lectin or antibodies. In this study, we attempted to destroy the endogenous *GGTA1* that synthesizes the α-Gal epitope [47] that is easily detectable by staining cells/embryos with fluorescence-labeled IB4 lectin [41,48]. Therefore, this detection system, based on the loss of cell surface α-Gal epitope expression, enables us to assess the effects of CRISPR/Cas9-mediated genome editing at earlier embryonic developmental stages.

In our previous study using cloned porcine blastocysts reconstituted with biallelic KO embryonic fibroblasts, complete loss of α-Gal epitope expression was observed in those embryos after fluorescence (AF594)-labeled IB4 staining [27]. Therefore, it is easy to speculate that the offspring cells of a blastocyst derived from an oocyte cytoplasmically injected with gRNA (targeted to *GGTA1*) and Cas9 mRNA might harbor a biallelic KO phenotype, and exhibit loss of α-Gal epitope expression on their surface. Unfortunately, all of the resulting blastocysts exhibited α-Gal epitope expression throughout the entire embryo, although some portions of the embryos exhibited reduced α-Gal epitope expression (see arrows in Figure 2). This suggests that porcine blastocysts resulting from oocyte microinjection frequently exhibit mosaicism with respect to mutated cells. This is also supported by the distribution of co-introduced *EGFP* mRNA in blastocysts. Almost all of the fluorescent embryos exhibited a mosaic expression of this protein (left panel of Figure 2A). Sequencing analysis also confirmed this mosaicism since the fluorescent blastocyst no. 1 had at least three types of *GGTA1* alleles, two mutated alleles (Figure 4B), and a wild-type allele (lower panel of Figure 3).The other fluorescent blastocyst, no. 10, had at least two types of *GGTA1* alleles, one mutated allele (Figure 4B) and a wild-type allele (lower panel of Figure 3).

The frequent mosaicism observed in our system might be caused by the poor spreading of the introduced mRNA throughout the egg cytoplasm, which might have resulted in asymmetric mRNA accumulation leading to mosaicism. However, our preliminary observations did not support this speculation because almost all two-cell/four-cell stage porcine embryos derived from the PA oocytes injected with CRISPR/*Cas9*-related mRNA and *EGFP* mRNA exhibited relatively uniform EGFP expression (unpublished results). Therefore, it is possible that *EGFP* mRNA segregation (probably CRISPR/*Cas9*-related mRNA segregation, too) might occur in later developmental stages, probably during the four-cell to morula stages. The presence of at least two types of cell populations (biallelic and monoallelic KO cells) in a blastocyst suggests that CRISPR/Cas9-mediated indel mutations might occur in later preimplantation developmental stages. Alternatively, the degree of mutation induction by the CRISPR/Cas9 system might be different in each blastomere of two-cell to four-cell stage embryos, leading to the generation of offspring cells with various mutation types. It may be reasonable to consider that the Cas9 synthesized from the injected mRNA remains active throughout several cell divisions in early embryo development, which may have triggered different mutations in different blastomeres, thus leading to mosaic embryos. Notably, Zhu *et al.* [21] demonstrated that 16 out of 19 newborn F0 mice obtained after zygote injection of gRNA (targeted to *Them2*) and Cas9 mRNA had indel mutations, and all (7/7) samples tested had one or more mutations in addition to the wild-type allele, suggesting a high degree of mosaicism. In this regard, it would be interesting to examine how indel mutations generated from the zygote to the cleavage stages are transmitted to the offspring cells of a blastocyst.

Our present T7E1 assay-based analysis for detection of indel mutations demonstrated that of the 16 samples tested (10 fluorescent and six non-fluorescent blastocysts), six (five fluorescent and one non-fluorescent) blastocysts had indel mutations (Figure 3; Table 1), indicating a mutation induction rate of 38% (6/16). Our present mutation induction rate in porcine zygotes appears to be lower than the very high mutation induction rates (67%–100%) already achieved in pigs [28] and other animals such as mice [17,39].

In this study, we employed WGA to amplify the whole genome from a single blastocyst. WGA is now recognized as an efficient and powerful method for genomic DNA amplification from organisms/cells with a very small amount of DNA in an unbiased manner [49]. In this case, the most important problem associated with this technology would be whether the target DNA can be amplified reproducibly avoiding biased amplification. In this regard, we have previously succeeded in amplifying target genes (*EGFP*) exogenously introduced into porcine cells together with the endogenous *Nanog* and *GGTA1* [42]. In addition, in this study, we have successfully amplified endogenous genes recognized as candidate genes (zinc finger protein *GLIS3*, unknown, and *dynamin-3-like*) for off-target analysis (see Figure 5). Notably, Sakurai *et al.* [39] compared the quality between DNA samples amplified using WGA and those amplified using repeated PCR from the same single blastocyst source, and found that there was no qualitative difference since several target genes were successfully amplified using both methods. Thus, we believe that WGA is a useful technique, especially when multiple analyses are required using samples with very small amounts of DNA.

We demonstrated the usefulness of a single blastocyst-based assay in pigs when zygote injection of CRISPR/Cas9-related components is applied. This approach is especially beneficial for testing the feasibility of several candidate gRNAs that are targeted to different portions of a target gene in a relatively short period (within seven days after PA), before transferring the genome-edited embryos to surrogate mothers. After this screening, it is possible to perform zygote injection using the selected gRNA for producing genome-edited piglets.

In future works, two issues will need to be considered: (1) determination of the appropriate mRNA concentration that confers efficient KO of a target gene upon oocyte injection experiments, and (2) determination of the suitable embryonic stage to achieve the best KO efficiency in porcine embryos. As for the former issue, here we used a RNA concentration that is commonly used for KO mice production. This concentration does not seem to be harmful for porcine embryonic development, but often causes mosaicism, as mentioned above. It is expected that an increased mRNA concentration (approximately three-fold for each mRNA) could increase KO efficiency and reduce mosaicism. However, this attempt failed to reduce mosaicism (unpublished results). As for the latter issue, it is possible that different injection timings might affect KO efficiency. In order to investigate this, we are currently carrying out injection experiments immediately (as shown in this study) or 6 h (at a stage just before the cleavage into two-cell embryos) after electric activation using porcine parthenotes. As a result, we found that injection 6 h after electric activation resulted in an elevated expression of the exogenous mRNA (unpublished results).

## 3. Experimental Section

### 3.1. In Vitro Synthesis of Cas9 mRNA, gRNA and EGFP mRNA

All CRISPR/Cas9 components were the same as reported in our previous paper [27]. For *Cas9* expression in mammalian cells, we used a plasmid encoding the human *Cas9*, a gift from George Church (Addgene plasmid #41815) [33]. The plasmid for gRNA#3 was used to target a sequence spanning the ATG in exon 4 of *GGTA1* (Figure S1). The *Cas9* coding sequence and the *EGFP* cDNA in pEGFP-N1 (Invitrogen, Carlsbad, CA, USA) with poly(A) sequence were cloned into the pBluescript II plasmid, which harbored a T7/T3 promoter for *in vitro* transcription.

In order to prepare *in vitro*-synthesized *Cas9* and *EGFP* mRNA, the constructed plasmids were linearized by *Sap* I and *in vitro* transcribed by T3 mMESSAGE mMACHINE kit (#AM1348; Ambion, Austin, TX, USA) following the manufacturer’s instructions. The prepared RNAs were purified using MEGAclear kit (#AM1908; Ambion) and recovered in RNase-free HEPES-buffered saline (#H3537; Sigma-Aldrich Co., St. Louis, MO, USA). The gRNA#3 plasmid was PCR-amplified with α3GalTt7-SF (5ʹ-agtaatacgactcactatagggagaaaataatgaatgtc-3ʹ) and gRNA-SR (5ʹ-aaaaaaagcaccgactcggtgccactttttcaagt-3ʹ) using KOD-plus (#KOD-201; Toyobo Co., Osaka, Japan). The PCR product was checked by electrophoresis on agarose gel and purified by ethanol precipitation. The synthetic gRNA was transcribed by T7 mMESSAGE mMACHINE kit (#AM1347; Ambion) from the template. Finally, all the RNA products were checked by electrophoresis on 2% agarose gel containing formalin using 3-(*N*-morpholino)propanesulfonic acid (MOPS) buffer prior to the microinjection.

### 3.2. Production of PA Embryos and Cytoplasmic Microinjection of RNAs

We used similar methods to those described in our previous studies for the production of porcine PA embryos [50]. Briefly, ovaries collected from prepubertal gilts at a local slaughter house were transported to the laboratory. Cumulus oocyte complexes (COCs) were extracted from 2- to 5-mm diameter antral follicles using an 18-gauge needle (Terumo, Tokyo, Japan) fixed to a 5-mL disposable syringe (Nipro, Osaka, Japan). The COCs were washed three times with HEPES (Nacalai Tesque, Kyoto, Japan) buffered Tyrode’s lactate-pyruvate-polyvinyl alcohol (PVA; Sigma-Aldrich Co.) (hereafter referred to as HEPES-TLP-PVA). Next, approximately 40–50 COCs were transferred to 200 μL maturation medium (90% (*v*/*v*) TCM-199 with Earle’s salts; Gibco BRL, Grand Island, NY, USA) supplemented with 0.91 mM sodium pyruvate (Sigma-Aldrich Co.), 3.05 mM d-glucose (Wako Pure Chemical, Osaka, Japan), 0.57 mM cysteine hydrochloride hydrate (Sigma-Aldrich Co.), 10 ng/mL epidermal growth factor (Sigma-Aldrich Co.), 10 IU/mL eCG (Aska Pharmaceutical Co., Tokyo, Japan), 10 IU/mL hCG (Aska Pharmaceutical Co.), 100 µg/mL amikacin sulfate (Meiji Seika, Tokyo, Japan), 0.1% (*w*/*v*) PVA, and 10% (*v*/*v*) pig follicular fluid that had been covered with paraffin oil (Nacalai Tesque) in a 35-mm dish (#1008; Becton Dickinson, Franklin Lakes, NJ, USA), and pre-equilibrated at 38.5 °C in an atmosphere of 5% CO_2_ overnight. After 42 to 44 h of maturation, cumulus cells were removed by pipetting with 0.1% (*w*/*v*) hyaluronidase (Sigma-Aldrich Co.). Oocytes with a polar body were selected for the experiments.

For PA embryo production, denuded oocytes (20–40) were placed between two wire electrodes 1 mm apart in an activation medium (250.3 mM sorbitol, 0.5 mM Ca(CH_3_COO)_2_, 0.5 mM Mg(CH_3_COO)_2_, and 0.1% BSA) [44]. Activation was induced with one direct current pulse of 100 V/mm for 50 μs using a LF101 Fusion Machine (Nepa Gene Co., Chiba, Japan). Activated oocytes were transferred to HEPES-TLP-PVA and then subjected to a single 2-pL cytoplasmic injection of 120 ng/μL human *Cas9* mRNA, 50 ng/μL gRNA, and 120 ng/μL *EGFP* mRNA. Injection success was confirmed by the slight oocyte cytoplasm swelling. The injected oocytes were then cultured in a drop (50 μL) of modified PZM-3 (mPZM-3) medium [51] containing 0.5 μM latrunculin A (LatA) (# L5163; Sigma-Aldrich Co.) for 2 h at 38.5 °C to increase their *in vitro* developmental rate [44]. After washing with mPZM3 medium three times, these oocytes were cultured in mPZM3 medium under an atmosphere of 5% CO_2_:5% O_2_:90% N_2_ at 38.5 °C. Cleavage and blastocyst formation were evaluated after 2 and 7 days of culture, respectively. The developing blastocysts were then fixed for 5 min at room temperature in 4% PFA in phosphate-buffered saline, pH 7.4 (PBS), prior to observation of fluorescence and subsequent lysis as described below.

### 3.3. T7E1-Based Assay and Sanger Sequencing of Mutated Sites

The single fixed blastocyst was transferred to a PBS drop (1 μL) in a 0.5-mL PCR tube (#PCR-05-C.; AxyGen Scientific, Inc., Union City, CA, USA) with the aid of a mouthpiece-controlled micropipette. Genomic DNA was extracted by adding 20 μL of lysis buffer (comprising 0.125 μg/mL of proteinase K, 0.125 μg/mL of Pronase E, 0.32 M sucrose, 10 mM Tris-HCl (pH 7.5), 5 mM MgCl_2_, and 1% (*v*/*v*) Triton X-100) onto the blastocyst-containing drop and incubating for 2–3 days at 37 °C, followed by phenol/chloroform extraction. Next, the supernatant was ethanol-precipitated with the aid of a GenTLE^®^ Precipitation Carrier (#9094; Takara Bio, Inc., Ohtsu, Japan). The precipitated DNA was dissolved in 20 μL of sterile water and stored at 4 °C.

In order to increase the total genomic DNA amount, we employed WGA using the illustra GenomiPhi V2 DNA Amplification kit (#25-6600-31; GE Health Care Japan, Tokyo, Japan) as previously described [42]. Briefly, 2 μL of genomic DNA was mixed with 8 μL of reaction buffer containing the enzyme in a volume of 20 μL and then incubated overnight at 30 °C. The resulting WGA products (2 μL) were first subjected to the first PCR round, using the Ex-S and Ex-RV primer set (Table S1 and Figure S1) in a volume of 20 μL and the PCR conditions previously described [27]. As controls, genomic DNA (~5 ng) isolated from normal PEFs [27] were concomitantly PCR-amplified. Next, nested PCR was performed using 2 μL of the first PCR products, primer sets Ex4-2S and Ex4-2RV (Table S1 and Figure S1) in a volume of 20 μL, and the same PCR conditions as those used in the first PCR round. The size of the resulting product was ~350 bp. In some cases, nested PCR was performed using 2 μL of the first PCR products and the primer sets N-S and D-RV (Table S1 and Figure S1) in a volume of 20 μL to detect the sequence corresponding to the *GGTA1* wild-type allele. The size of the resulting product was 95 bp. One microliter of these products was then subjected to electrophoresis in 2% agarose gels for checking the band size. The remaining products were then ethanol-precipitated, re-suspended in ~20 μL of sterile water, and the DNA content was measured using a spectrophotometer.

For the T7E1-based cleavage assay, 10 μl of 1× NEB2 reaction buffer (New England BioLabs Japan Inc., Tokyo, Japan) containing 400 ng of the nested PCR products derived from the experimental sample (200 ng; derived from a blastocyst developed from a mRNA-injected oocyte) and the control sample (200 ng; derived from normal PEFs) were placed in a 0.5-mL PCR tube (AxyGen Scientific, Inc.). The PCR tube was incubated at 95 °C for 5 min for denaturation using a thermal cycler (PC-708; Astec, Fukuoka, Japan) and then incubated for 0.5–1 h at room temperature (24 °C) for re-annealing to generate heteroduplex DNA. Next, 1 μL of T7E1 (2.5 U/μL; New England BioLabs Japan Inc.) was added to the denatured/re-annealed sample and the PCR tube was incubated at 37 °C for 1 h. The nuclease-treated solutions were electrophoresed in a 2% agarose gel, stained with ethidium bromide, and photographed under ultraviolet illumination.

For sequencing, some of the nested PCR products of ~350 bp size were first subjected to direct sequencing using the E-S primer set (Table S1 and Figure S1). Furthermore, some of these PCR products were subcloned into the TA cloning vector pCR2.1 (Invitrogen) for sequencing. The resulting clones were sequenced using the E-S primer set. Direct sequencing using dye termination cycle sequencing was performed at FASMAC Co., Ltd. (Atsugi, Kanagawa, Japan).

### 3.4. Screening and Detection of Off-Target Sites

The potential off-target sites were identified based on the internet web URL (http://crispr.mit.edu). From the candidates with the highest scores, three genes were selected (Table S2). In order to amplify these genes, the WGA products (2 μL) derived from several blastocyst samples were first PCR-amplified using different primer sets (#1, #2 and #3 products were amplified with 1-S and 1-RV, 2-S and 2-RV, and 3-S and 3-RV, respectively; Table S1) in a 20-μL volume, using the previously described PCR conditions [27]. A nested PCR was next performed using the first PCR products (2 μL) and different primer sets (#1, #2 and #3 products were amplified with 1-2S and 1-2RV, 2-2S and 2-2RV, and 3-2S and 3-2RV, respectively; Table S1) in a volume of 20 μL, using the same PCR conditions used for the first PCR. After checking a portion of the nested PCR products, they were ethanol-precipitated and, finally, dissolved in 20 μL of sterile water. Then, the T7E1 assay was carried out as described above to confirm whether off-target mutations existed.

### 3.5. Staining with AF594-IB4 and Fluorescence Detection

Staining of embryos with AF594-IB4 (#I21413; Invitrogen) was described in a previous article [27]. The stained embryos were examined under a fluorescence microscope (BX60; Olympus, Tokyo, Japan) using DM505 (BP460-490 and BA510IF; Olympus) and DM600 filters (BP545-580 and BA6101F; Olympus), which were used for detecting EGFP-derived green fluorescence and AF594-derived red fluorescence, respectively. Micrographs were taken using a digital camera (FUJIX HC-300/OL; Fuji Film, Tokyo, Japan) attached to the fluorescence microscope, and images were printed using a Mitsubishi digital color printer (CP700DSA; Mitsubishi, Tokyo, Japan).

## 4. Conclusions

In this study, we succeeded in inducing indel mutations in an endogenous target locus using cytoplasmic injection of CRISPR/Cas9-related mRNA into porcine PA oocytes with a ~38% efficiency. However, almost all of the developing blastocysts exhibited mosaicism upon mutation induction. The development of a technique to avoid or reduce such mosaicism would be one of the key factors that will allow efficient KO piglet production by zygote injection.

## Supplementary Materials

Supplementary materials can be found at http://www.mdpi.com/1422-0067/16/08/17838/s1.
