# Supplementary material for: Direct Injection of CRISPR/Cas9-Related mRNA into Cytoplasm of Parthenogenetically Activated Porcine Oocytes Causes Frequent Mosaicism for Indel Mutations
Source: Int J Mol Sci. 2015 Aug 3;16(8):17838–56. doi: 10.3390/ijms160817838 (PMC4581225; doi:10.3390/ijms160817838)
Supplement: Supplementary file 1 [file ijms-16-17838-s001.pdf]

Supplementary Information

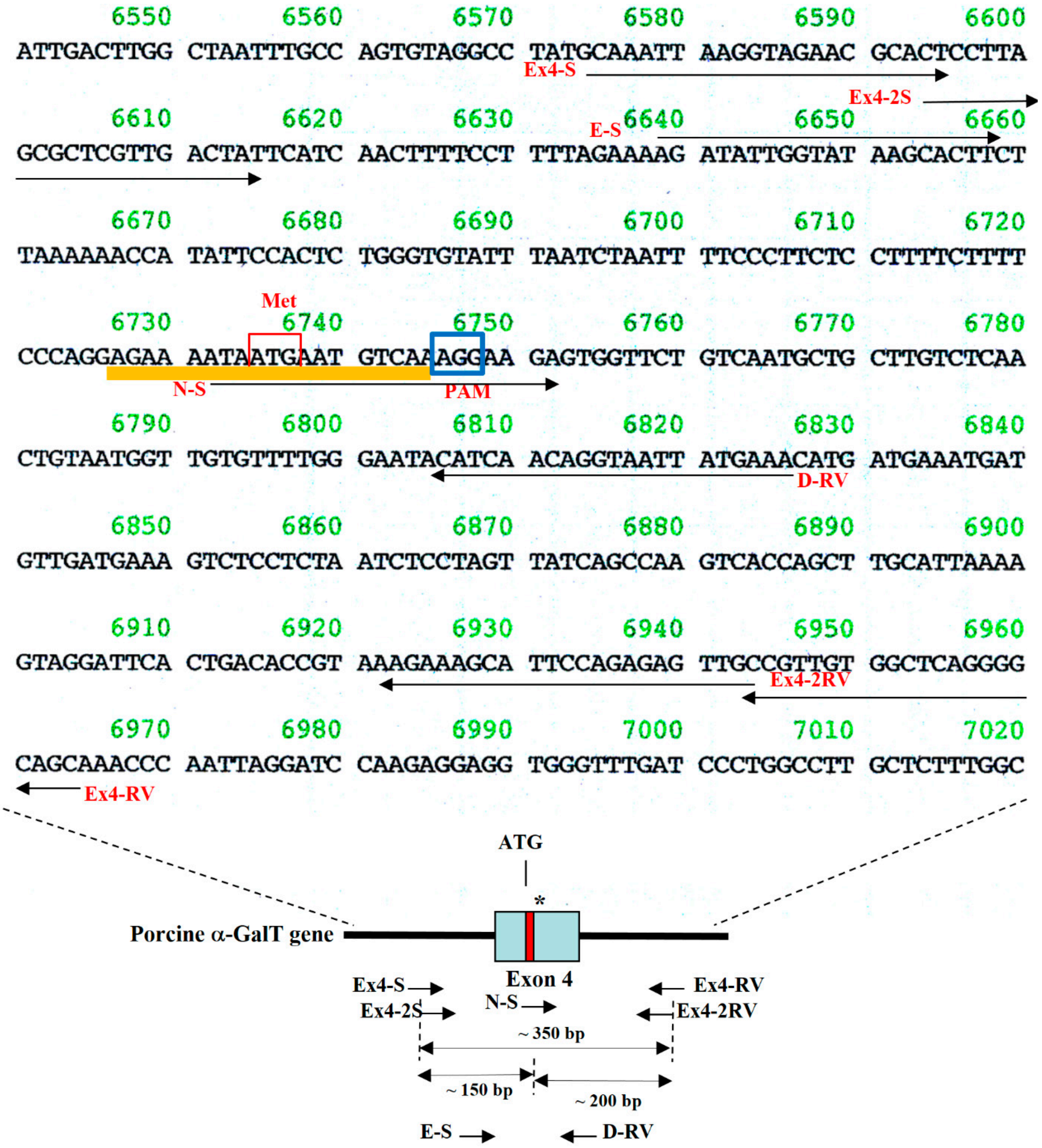

Figure S1. Genomic sequence spanning exon 4 of porcine *GGTA1*. The translation innitiation site, ATG, is shown in a red box. The PAM (protospacer adjacent notif) site is shown in a blue box. The target sequence recognized by gRNA#3 is shown underlined in orange. Primers used for PCR and sequencing are indicated by arrows underneath the sequence and in a schematic representation of the exon 4 of *GGAT1*. Asterisks indicate the mutated portion of the exon 4 of *GGAT1*.

Table S1. Primer sets used for PCR.

Primer	Sequence (5′–3′)	Reference
N-S	ATAATGAATGTCAAAGGAAGA	–
D-RV	TTTCATAATTACCTGTTGATG	–
E-S	AGATATTGGTATAAGCACTTC	–
Ex4-S	GCAAATTAAGGTAGAACGCA	[27]
Ex4-2S	CTCCTTAGCGCTCGTTGGCT	[27]
Ex4-RV	GCTGCCCCTGAGCCACAACG	[27]
Ex4-2RV	GCAACTCTCTGGAATGCTTT	[27]
1-S	AGAGGGTAACATTTGAGTAGA	–
1-2S	TAACATTTGAGTAGAGACCTT	–
1-RV	TTCAAAATCTATGTTACTGGT	–
1-2RV	ATCTATGTTACTGGTTCATGA	–
3-S	CCTACACCACAGCCACAGCAA	–
3-2S	CCACAGCCACAGCAACATGGG	–
3-RV	GGAGGGAGGCTGCAGTAGAAA	–
3-2RV	AGGCTGCAGTAGAAATAGGAA	–
4-S	TAACCCACTGCACTGAGCTGG	–
4-2S	ACTGCACTGAGCTGGGGACTG	–
4-RV	ATGTGTTCAATAAATATAAGA	–
4-2RV	TCAATAAATATAAGATGAATA	–

Table S2. List of candidate genes that potentially cause off-target mutations.

Sequence	Score	Mismatches	Locus
(1) GAAAAAATAATGAATGACAAGAG [1]	4.6	2MMs [3:17]	chr1: −243831704
(2) GAGAAAAAAATGAATGTCATGGG [2]	4.2	2MMs [8:20]	chr940: +158850
(3) GATAAAATAATGAATGTAAAAGG [3]	2.6	2MMs [3:18]	chr9: −126332949

[1] The gRNA#3 used here exhibits similarity to *Sus scrofa* breed mixed chromosome 1, Sscrofa10.2 zinc finger protein GLIS3 (Sequence ID: ref|NC_010443.4| Length: 315321322 Number of Matches: 3076), as shown below.

```
Query  1          GAAAAAATAATGAATGACAAGAG  23
                  |||||||||||||||||||||||
Sbjct  243831731  GAAAAAATAATGAATGACAAGAG  243831709
```

[2] The gRNA#3 used here also exhibits similarity to *Sus scrofa* breed mixed unplaced genomic scaffold, Sscrofa10.2 ChrUScaf1915 (Sequence ID: ref|NW_003538807.1| Length: 253766 Number of Matches: 4), as shown below.

```
Query  1       GAGAAAAAAATGAATGTCATGGG  23
               |||||||||||||||||||||||
Sbjct  158852  GAGAAAAAAATGAATGTCATGGG  158874
```

[3] The gRNA#3 used here also exhibits similarity to *Sus scrofa* breed mixed chromosome 9, Sscrofa10.2 dynamin-3-like (Sequence ID: ref|NC_010451.3| Length: 153670197 Number of Matches: 1149), as shown below.

```
Query  1          GATAAAATAATGAATGTAAAAGG  23
                  |||||||||||||||||||||||
Sbjct  126332976  GATAAAATAATGAATGTAAAAGG  126332954
```
